# 690. The New Kid On The Block: Use of Maribavir in Pediatric Immunocompromised Hosts

**DOI:** 10.1093/ofid/ofaf695.229

**Published:** 2026-01-11

**Authors:** Antigone Kraft, Madeline Johnson, Gabriela Maron, Diego R Hijano, William R Otto, Lara A Danziger-Isakov, Tanvi S Sharma, Surabhi Vora, Kari Neemann, Claire Bocchini, Joana Dimo, Benjamin Hanisch

**Affiliations:** Food and Drug Administration, Silver Spring, MD; St. Jude Children's Research Hospital, Memphis, Tennessee; St. Jude Children's Research Hospital, Memphis, Tennessee; St. Jude Children's Research Hospital, Memphis, Tennessee; Cincinnati Children's Hospital Medical Center, Cincinnati, OH; Cincinnati Children's Hospital, Cincinnati, OH; Boston Children's Hospital, Boston, MA; Seattle Children's Hospital/University of Washington, Seattle, Washington; University of Nebraska Medical Center - Children's Nebraska, Omaha, Nebraska; Baylor College of Medicine, Houston, Texas; University of Colorado/Children's Hospital Colorado, Denver, Colorado; Children’s National Hospital, Washington, DC, USA, Washington, District of Columbia

## Abstract

**Background:**

Immunocompromised patients with Cytomegalovirus (CMV) infections may experience a variety of clinical syndromes ranging from graft dysfunction to death. Standard treatment options are often limited by end-organ toxicities and resistant/refractory CMV disease. Maribavir (MBV) is currently FDA-approved for the treatment of CMV infection that is refractory to other antivirals in patients at least 12 years of age and weighing at least 35 kg, but there is a paucity of data in younger populations. Despite this, MBV is used off-label due to its activity against resistant CMV, oral formulation and mild side effect profile compared to other agents.

**Figure 1:**
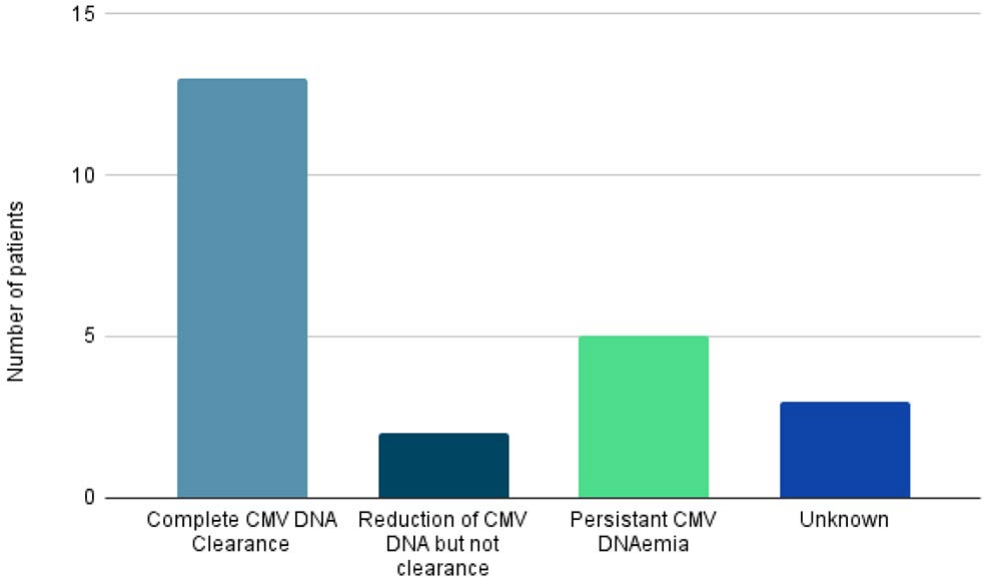
Participant Outcomes after Completion of Maribavir Therapy

**Table 1:**
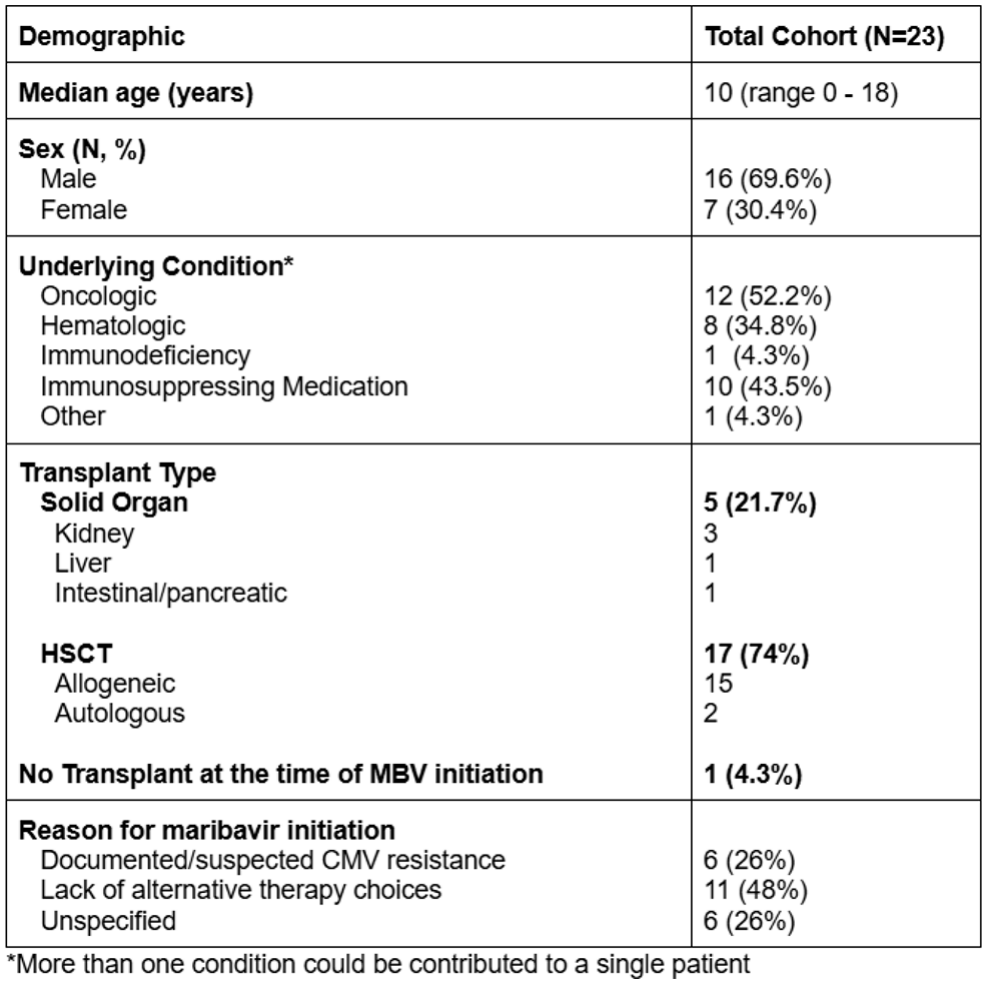
Demographic Data

**Methods:**

This is an ongoing, multicenter, retrospective study examining the clinical characteristics and outcomes of pediatric immunocompromised patients who received MBV therapy. Inclusion criteria included patients (age < 18 years) with documented CMV DNAemia, who received chimeric antigen receptor T-cell (CAR-T), solid organ transplantation, or hematopoietic stem cell transplantation therapies. Cases were assembled from participating institutions within the Pediatric Infectious Diseases Transplant Network (PIDTRAN).

**Results:**

Of available data, 23 patients from 7 institutions received at least one dose of MBV (median age 10 years). The majority of participants were male (16/23, 69%). Most patients had an underlying oncologic diagnosis and were undergoing HSCT. Eleven (48%) patients initiated MBV due to the lack of alternative therapy choices (Table 1). Complete resolution of CMV DNAemia was achieved in 13/23 (56.5%) patients. CMV DNAemia control as noted by a reduction in viremia without complete clearance was also noted. Persistence of CMV DNAemia was reported in 5/23 (22%), and resistance to MBV was confirmed in one patient (Figure 1). No patients discontinued MBV due to adverse events, with only one patient reporting drug-related dysgeusia.

**Conclusion:**

We present the largest retrospective case series in pediatrics on MBV use in immunocompromised hosts. MBV is effective in treating CMV DNAemia, well tolerated, and minimal resistance is noted. This study is ongoing and updated data will be presented. A prospective study is needed to definitively assess the safety and efficacy of this drug in pediatrics.

**Disclosures:**

Lara A. Danziger-Isakov, MD, MPH, AiCuris.: Grant/Research Support|Ansun BioPharma: Grant/Research Support|Astellas Pharma Global Development, Inc: Advisor/Consultant|Astellas Pharma Global Development, Inc: Grant/Research Support|kamada: Advisor/Consultant|Merck Sharp & Dohme Corporation: Grant/Research Support|Pfizer (Any division): Grant/Research Support|Takeda Pharmaceuticals: Grant/Research Support

